# *In Vivo* Persistence of Chimeric Virus after Substitution of the Kaposi's Sarcoma-Associated Herpesvirus LANA DNA Binding Domain with That of Murid Herpesvirus 4

**DOI:** 10.1128/JVI.01251-18

**Published:** 2018-10-12

**Authors:** Marta Pires de Miranda, Ana Patrícia Quendera, Colin E. McVey, Kenneth M. Kaye, J. Pedro Simas

**Affiliations:** a Instituto de Medicina Molecular, Faculdade de Medicina, Universidade de Lisboa, Lisbon, Portugal; b Instituto de Tecnologia Química e Biológica António Xavier, Universidade Nova de Lisboa, Oeiras, Portugal; c Departments of Medicine, Brigham and Women's Hospital and Harvard Medical School, Boston, Massachusetts, USA; University of Southern California

**Keywords:** Kaposi's sarcoma-associated herpesvirus, LANA, murid herpesvirus 4

## Abstract

The latency-associated nuclear antigen from Kaposi's sarcoma-associated herpesvirus (KSHV), kLANA, and its homolog from the murid herpesvirus 4 (MuHV-4), mLANA, are essential for viral latency. kLANA is nearly four times the size of mLANA, mainly due to an extensive central repeat region that is absent in mLANA. Both proteins harbor a C-terminal DNA binding domain (DBD). The DBD binds the terminal repeat (TR) DNA sequences of the viral genome to mediate persistence. Despite structural conservation, the kLANA and mLANA DBDs differ in sequence and mode of oligomerization. kLANA DBD oligomers are flexible and bent, while mLANA DBD oligomers bind DNA in a rigid, linear conformation. We previously reported that kLANA and mLANA acted reciprocally on TR sequences. Furthermore, a MuHV-4 expressing kLANA instead of mLANA (v-kLANA) established latency in mice, albeit at a lower magnitude than the wild-type (WT) virus. Here, we asked if kLANA can accommodate the mLANA DBD and generated a fusion protein which contains kLANA but with the mLANA C-terminal region in place of that of kLANA. We report a recombinant MuHV-4 (v-KM) encoding this LANA fusion protein instead of mLANA. The fusion protein was expressed in lytic infection *in vitro* and assembled nuclear LANA dots in infected splenocytes. Results demonstrated that kLANA functionally accommodated mLANA's mode of DNA binding, allowing MuHV-4 chimeric virus to establish latency *in vivo*. Notably, v-KM established latency in germinal center B cells more efficiently than did v-kLANA, although levels were reduced compared to WT MuHV-4.

**IMPORTANCE** KSHV is a human oncogenic virus for which there is no tractable, immunocompetent animal model of infection. MuHV-4, a related rodent gammaherpesvirus, enables pathogenesis studies in mice. In latency, both viruses persist as extrachromosomal, circular genomes (episomes). LANA proteins encoded by KSHV (kLANA) and MuHV-4 (mLANA) contain a C-terminal DNA binding domain (DBD) that acts on the virus terminal repeats to enable episome persistence. mLANA is a smaller protein than kLANA. Their DBDs are structurally conserved but differ strikingly in the conformation of DNA binding. We report a recombinant, chimeric MuHV-4 which contains kLANA in place of mLANA, but in which the DBD is replaced with that of mLANA. Results showed that kLANA functionally accommodated mLANA's mode of DNA binding. In fact, the new chimeric virus established latency *in vivo* more efficiently than MuHV-4 expressing full-length kLANA.

## Abstract (precis)

KSHV is a human oncogenic virus for which there is no tractable, immunocompetent animal model of infection. MuHV-4, a related rodent gammaherpesvirus, enables pathogenesis studies in mice. In latency, both viruses persist as extrachromosomal, circular genomes (episomes). LANA proteins encoded by KSHV (kLANA) and MuHV-4 (mLANA) contain a C-terminal DNA binding domain (DBD) that acts on the virus terminal repeats to enable episome persistence. mLANA is a smaller protein than kLANA. Their DBDs are structurally conserved but differ strikingly in the conformation of DNA binding. We report a recombinant, chimeric MuHV-4 which contains kLANA in place of mLANA, but in which the DBD is replaced with that of mLANA. Results showed that kLANA functionally accommodated mLANA's mode of DNA binding. In fact, the new chimeric virus established latency *in vivo* more efficiently than MuHV-4 expressing full-length kLANA.

## INTRODUCTION

The Kaposi′s sarcoma-associated herpesvirus (KSHV or human herpesvirus 8) is a human pathogen with etiologic roles in Kaposi′s sarcoma and several B cell lymphoproliferative disorders (1–3). KSHV belongs to the gamma-2 herpesvirus subfamily, as does the closely related murid herpesvirus 4 (MuHV-4 or MHV68), a rodent virus (4, 5). As with all herpesviruses, KSHV and MuHV-4 persist through the lifetime of the host, switching between lytic and latent infection. Unlike KSHV, MuHV-4 readily infects laboratory mice, providing a model to investigate gammaherpesvirus pathogenesis (6, 7). Both KSHV and MuHV-4 establish latency in B cells (8, 9). Inoculation of mice with MuHV-4 leads to self-limiting lytic replication followed by establishment and amplification of latency in germinal center (GC) B cells and long-term persistence in memory B cells (8, 10, 11).

In latency, the viral genome circularizes through fusion of its terminal repeat (TR) regions. The circular genome persists as a multicopy extrachromosomal episome (plasmid) in the nucleus (12, 13). Few viral genes are expressed in this phase of infection. Among these is the KSHV latency-associated nuclear antigen, kLANA, encoded by ORF73 (14, 15). kLANA from the prototype KSHV is 1,162 residues in length. The C-terminal region of kLANA harbors a conserved DNA binding domain (DBD) that associates with TR DNA sequences (16–19). kLANA associates with chromosomes via protein interactions, including association with histones H2A/H2B through the N-terminal region (20). Through these binding properties, kLANA tethers episomes to chromosomes, ensuring their segregation and persistence through mitosis (16, 21). kLANA also drives episome replication and modulates transcription and cell growth. kLANA is thus essential for KSHV latency.

The ORF73 of MuHV-4 encodes a kLANA homolog, mLANA, which is expressed in lytic and latent replication (10, 22–24). mLANA is essential for episome persistence and latency *in vitro* (25) and in infected mice (26–28). mLANA is smaller than kLANA, 314 versus 1,162 amino acid residues, lacking the extensive acidic and glutamine-rich internal repeat region of kLANA (residues 330 to 930). C-terminal mLANA has a DBD structurally similar to that of kLANA (29–31). Both kLANA and mLANA DBDs dimerize. Through interdimer interactions, both DBDs oligomerize and bind cooperatively to adjacent sites on TR DNA. However, mLANA oligomers are rigid and linear whereas kLANA oligomers are intrinsically bent, can adopt different angles of bend and twist, and induce bending of TR DNA upon binding (17, 19, 32). Therefore, kLANA and mLANA bind DNA in strikingly different ways.

We have shown binding of the kLANA DBD to MuHV-4 TR DNA and vice versa, from mLANA DBD to KSHV TR DNA (19, 24). Moreover, kLANA supported the persistence of plasmids containing MuHV-4 TR elements *in vitro* (24). With the aim of developing a mouse model to investigate kLANA functions *in vivo*, we and others constructed MuHV-4 encoding full-length kLANA instead of mLANA (24, 33). The chimeric viruses expressed the kLANA transgene and established latency in GC B cells, albeit at reduced levels compared to WT MuHV-4 (24, 33). In light of the different modes of DNA binding between kLANA and mLANA, we assessed here if kLANA can functionally accommodate the mLANA DBD. We generated a MuHV-4 expressing kLANA with the C-terminal region of mLANA instead of that of kLANA. We find that replacement of the kLANA DBD with that of mLANA results in a chimeric virus that establishes *in vivo* at enhanced levels compared to the kLANA chimeric virus.

## RESULTS

### Generation of MuHV-4 expressing kLANA-C-terminal mLANA fusion proteins.

**FIG 1:**
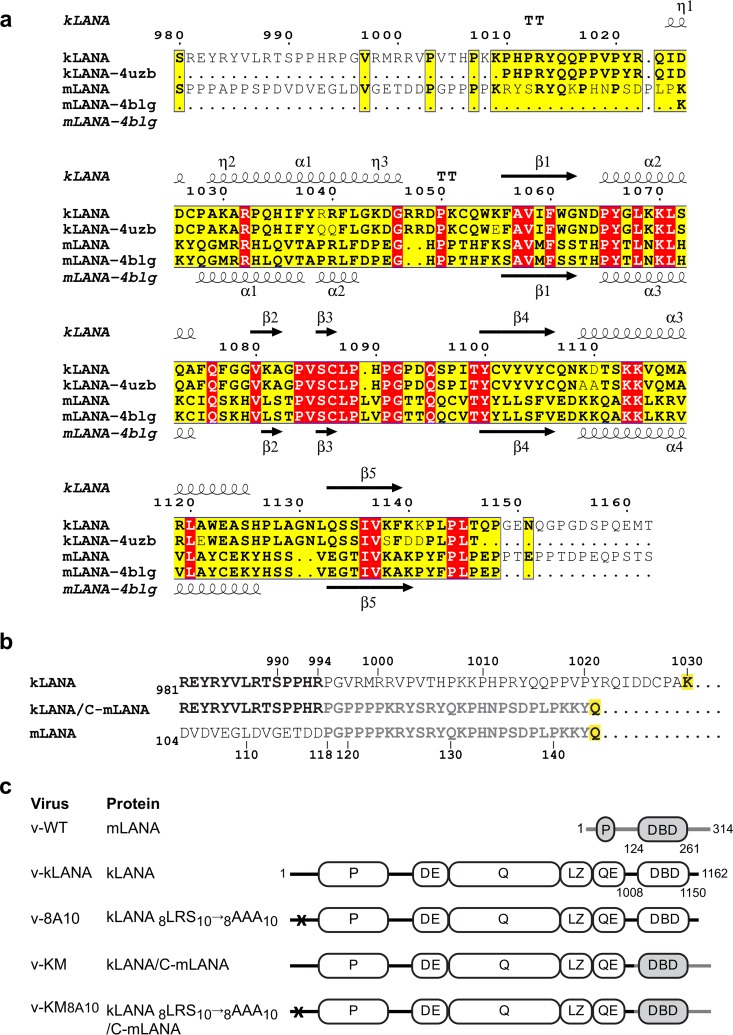
Construction of MuHV-4 expressing kLANA-C mLANA proteins. (a) Structure-based sequence alignment of the C-terminal regions of kLANA (accession number Q76SB0) and mLANA (accession number O41974) using Expresso (49) and ESPript 3.0 (50). Secondary structure elements of kLANA (PDB ID 4uzb) and mLANA (PDB ID 4blg) DBDs are shown above and below the alignments, respectively. Identical residues are highlighted in red, similar residues are highlighted in yellow. (b) Alignment of kLANA/C-mLANA fusion with kLANA residues 981 to 1030 and mLANA residues 104 to 144. Residues identical in kLANA and mLANA are in bold. Residues at the beginning of the α1 helix of the DBD are highlighted in yellow. (c) Proteins expressed by each virus. P, proline-rich region; DBD, DNA domain binding domain; DE, aspartate-glutamate; Q, glutamine; QE, glutamine-glutamate repeat regions; LZ, putative leucine zipper. mLANA is in gray. Crosses indicate mutations in the H2A/H2B binding site of kLANA. Numbers indicate amino acid residues.

**FIG 2:**
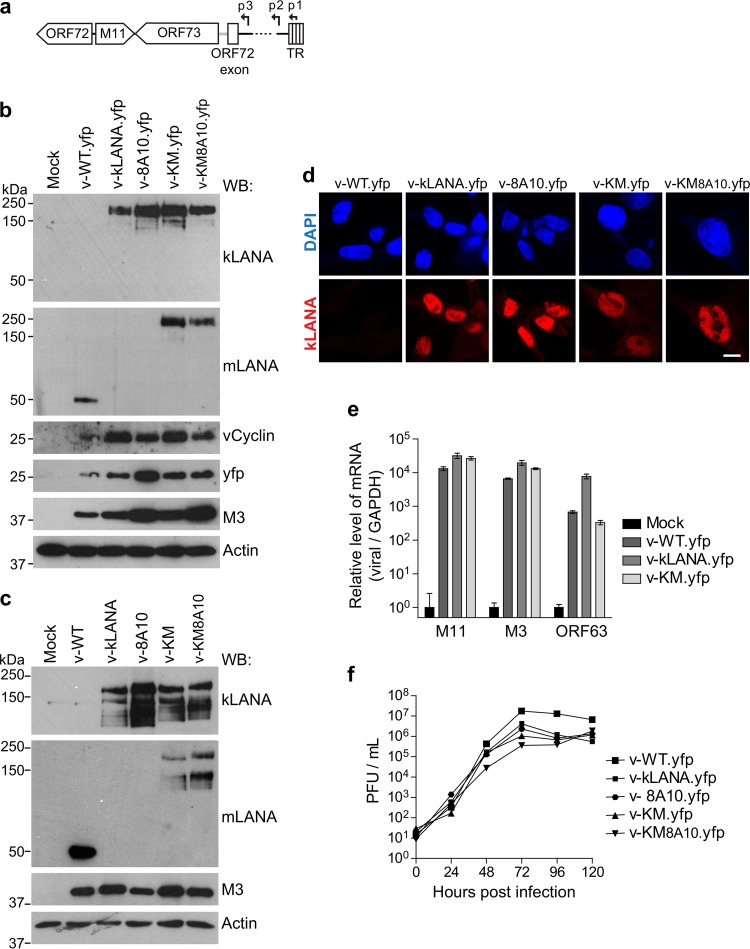
*In vitro* lytic infection. (a) kLANA MuHV-4 chimeras contain kLANA- or kLANA/C-mLANA-coding DNA inserted in the mLANA (ORF73) locus, in place of mLANA-coding DNA. Expression is driven by mLANA promoters p1, p2, and p3 (22, 23). TR, terminal repeats. (b, c) Detection of viral proteins in infected BHK-21 (3 PFU/cell, 6 h) cellular lysates using Western blotting (WB). kLANA was detected with MAb LN53, and mLANA was detected with MAb 6A3. Blotting against the MuHV-4 M3 protein and actin was used to assess levels of infection and loading, respectively. In panel b, expression of vCyclin and YFP was confirmed with anti-vCyclin and anti-GFP immunoblotting, respectively. (d) Images (confocal slices) depicting localization of LANA proteins in BHK-21 cells infected as described for panel a. DNA was stained with DAPI. Bar, 10 μm. (e) Relative levels (ΔΔ*C_T_*) of M11, M3, and ORF63 in infected BHK-21 cells (5 PFU/cell, 8 h) compared to uninfected cells, normalized to GAPDH. Bars indicate mean fold changes ± standard deviations (SD). (f) Growth curves in BHK-21 cells infected with 0.01 PFU/cell. Total virus titers were determined. Time zero indicates input of virus after washing inoculum. Virus titers did not differ significantly between infection groups (Kruskal-Wallis test).

To create a kLANA protein with the C-terminal region of mLANA in place of that of kLANA, we took into account a sequence alignment based on the solved structures of kLANA and mLANA DBDs (Fig. 1a) (29, 31). The aim was to preserve the structural integrity of the hybrid protein and maintain functional continuity between N-terminal kLANA and C-terminal mLANA. In the fusion protein, kLANA residues 1 to 994 were preserved and the C-terminal region (residues 995 to 1162) was replaced by the entire C-terminal mLANA (residues 118 to 314) (Fig. 1b and c). mLANA residues preceding the α1 helix of the DBD (Fig. 1a) were included, as they are required for efficient DNA binding (19). We also engineered a kLANA-C-mLANA fusion with mutations in the N-terminal H2A/H2B binding site (8LRS10 to 8AAA10) of kLANA (Fig. 1c) that abolish latency. Chimera MuHV-4 viruses encoding the fusion proteins in place of mLANA were termed v-KM and v-KM8A10 for the nucleosome binding mutant (Fig. 1c). As previously described, to generate MuHV-4 expressing kLANA (v-kLANA) or kLANA_8AAA10_ (v-8A10) (Fig. 1c) (24), DNA encoding the fusion proteins plus the kLANA 5′ untranslated region (UTR) was inserted into the mORF73 (mLANA) locus, between M11 and the mORF72 (vCyclin) exon (Fig. 2a). Expression of proteins is driven by mLANA promoters (22, 23) (Fig. 2a). Viruses were engineered in the genomes of WT MuHV-4 or in a MuHV-4 that expresses yellow fluorescent protein (YFP) (denoted by yfp).

### *In vitro* analysis of chimera viruses.

The kLANA-C-terminal mLANA fusion proteins were expressed during lytic replication *in vitro* (Fig. 2b to d). Monoclonal antibody 6A3 recognizes C-terminal mLANA. Monoclonal antibody LN53 recognizes EQEQ epitopes (34) present in the repeat regions comprising the leucine zipper (LZ) and the glutamine-glutamate (QE) repeats, just upstream of C-terminal kLANA (Fig. 1c). Since both of these regions are present in kLANA containing the mLANA DBD, both antibodies detected the fusion protein expressed in v-KM.yfp- and v-KM8A10.yfp-infected cells (Fig. 2b, top two panels) by Western blotting. mLANA in v-WT.yfp-infected cells and kLANA in v-kLANA.yfp-infected cells were also detected by monoclonal antibodies (MAbs) 6A3 and LN53, respectively (Fig. 2b, top 2 panels). Similar results were obtained in cells infected with the non-yfp versions of the viruses (Fig. 2c). mORF72, which is part of the same transcript as mORF73 (22), was expressed in all infected cell lysates (Fig. 2b, third panel from top). kLANA and fusion proteins localized to the nucleus of infected cells, with a broad distribution and some regions of more concentrated intensity (Fig. 2d). kLANA disengages from TR DNA during lytic infection, resulting in broad, nuclear distribution (35).

MuHV-4 M11 is a bcl-2 homolog required for efficient establishment of latency (36). M11 is transcribed in the opposite direction of mORF73 (Fig. 2a), and the M11 stop codon overlaps with the 3′ end of mORF73. We preserved the M11 stop codon in the chimera constructs. No differences were found in M11 mRNA levels between v-WT.yfp-, v-kLANA.yfp-, and v-KM.yfp-infected cells (Fig. 2e). Thus, phenotypes observed previously with v-kLANA (24) or here with C-terminal swap viruses are not due to altered M11 expression. In addition, M3 and ORF63 are lytic genes that encode a chemokine binding protein (10, 37, 38) and a tegument protein (39, 40), respectively. Neither M3 nor ORF63 mRNA levels were reduced in v-kLANA.yfp or v-KM.yfp compared to v-WT.yfp (Fig. 2e). All viruses grew similarly *in vitro* (Fig. 2f).

### v-KM establishes higher latency levels than v-kLANA.

To compare the pathogenesis of the kLANA-C-mLANA MuHV-4 chimera with the wild-type (WT) and the kLANA MuHV-4 chimera, we infected mice intranasally (i.n.) with 10^4^ PFU. Typically, at this inoculation dose, lytic virus titers in the lungs peak around day 7. Latent infection in the spleen peaks at around day 14, declining afterwards to very low or undetectable levels.

**FIG 3:**
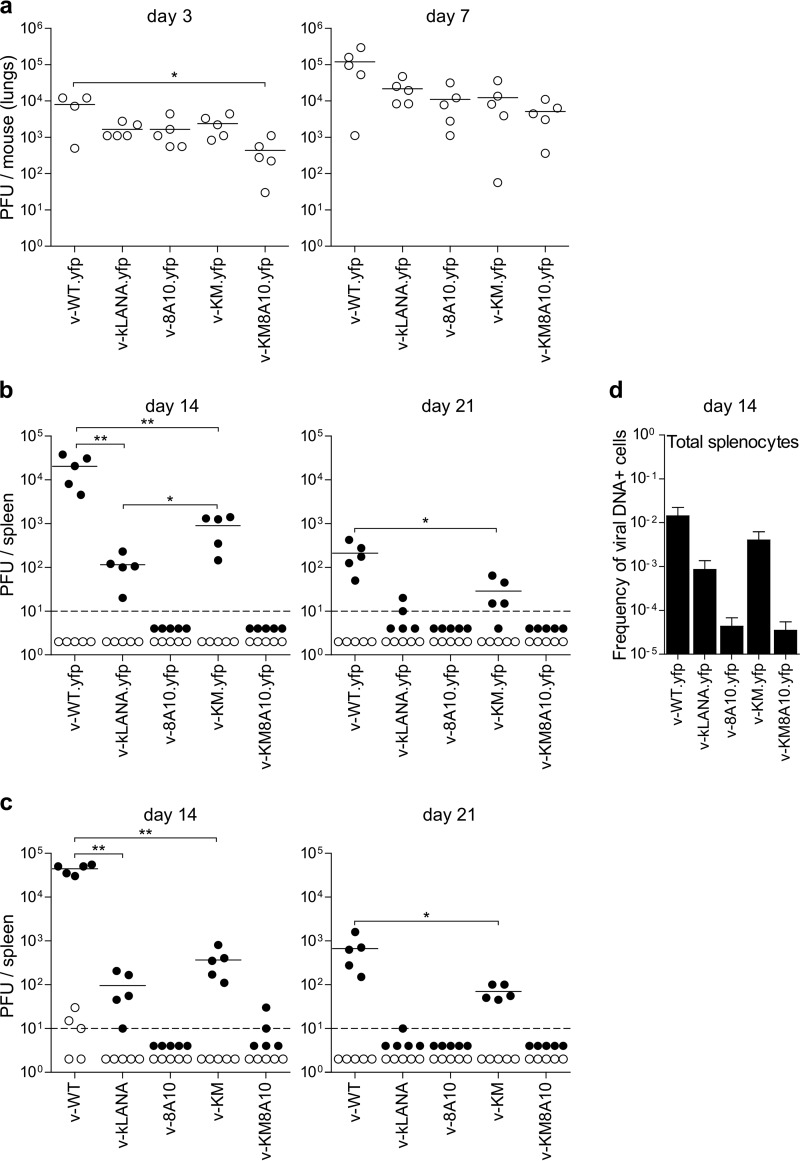
v-KM has higher latency loads than v-kLANA. C57 BL/6 mice were inoculated intranasally with 10^4^ PFU. (a) Lung virus titers at days 3 and 7 after infection. Circles represent individual mice. Horizontal bars indicate the means. v-WT.yfp had significantly higher titers than v-KM8A10.yfp at day 3 (*, *P* < 0.05, Kruskal-Wallis test followed by Dunn's multiple-comparison test). All other comparisons between infection groups were not significant (*P* > 0.05, Kruskal-Wallis test). (b, c) Infectious center/reactivating virus titers in spleens of mice infected with yfp (b) or non-yfp viruses (c). Black symbols, reactivating virus titers; open symbols, preformed infectious virus titers. Circles represent individual mice. Horizontal lines indicate the mean. The dashed line indicates the limit of detection. At day 14, WT viruses had significantly higher latency loads than did kLANA or KM viruses (**, *P* < 0.01 in panel b; *, *P* < 0.05 in panel c, Mann-Whitney test). Latency loads of KM viruses were significantly higher than those of kLANA viruses in panel b (*, *P* < 0.05, Mann-Whitney test) but not in panel c (*P* = 0.056, Mann-Whitney test). At day 21, WT viruses had higher latency loads than KM viruses (*, *P* < 0.05 in panels b and c, Mann-Whitney test). (d) Frequency of viral DNA^+^ cells in total splenocytes. Data are from pools of 5 mice per group. Error bars indicate 95% confidence intervals.

Lung virus titers were slightly reduced for all chimera viruses compared to wild-type virus (Fig. 3a). This reduction was less than 1 log at day 3 for all recombinants except for v-KM8A10.yfp, which had about 1 log reduction compared to v-WT.yfp (*P* < 0.05) (Fig. 3a, left panel). Differences of 0.5 to 1 log between average titers of the chimeric viruses compared to the WT group were also observed at day 7 but did not attain statistical significance (Fig. 3a, right panel).

To quantify latent virus, we performed *ex vivo* reactivation assays by coculturing total splenocytes with permissive BHK-21 cells. At day 14, reactivating viruses were detected in v-kLANA.yfp-infected mice but, as previously observed, were reduced significantly (*P* < 0.01) by nearly 2 log compared to v-WT.yfp infection (Fig. 3b, left panel). In contrast, v-KM.yfp-infected mice had a mean titer that was intermediate between that of v-kLANA.yfp and that of v-WT.yfp (Fig. 3b, left panel). At day 21, reactivating virus was clearly detectable in the v-KM.yfp infection group, with a mean titer 1 log lower than that of the WT group (*P* < 0.05) (Fig. 3b, right panel). In contrast, at this time after infection, v-kLANA.yfp reactivation titers were below or very near the limit of detection of the assay (Fig. 3b, right panel). v-8A10.yfp and v-KM8A10.yfp chimera viruses had no detectable reactivating virus anytime after infection (Fig. 3b). Preformed virus titers assessed in freeze-thawed samples were below or at the limit of detection. This confirms that coculture assay titers correspond to reactivation from latency (Fig. 3b). Similar results were obtained in *ex vivo* reactivation assays at days 14 and 21 after infection with the independent, non-yfp versions of the viruses (Fig. 3c).

We determined in parallel the frequency of infection in total splenocytes at day 14 by combining limiting dilution with PCR to detect viral genomes. The v-kLANA.yfp infection group had a 16-fold-lower frequency of infection than that of v-WT.yfp, which is similar to our previous results (24). v-KM.yfp displayed higher frequencies of infection than v-kLANA.yfp, which were closer but still 3.6-fold lower than WT levels (Fig. 3d and Table 1). Mutants v-8A10.yfp and v-KM8A10.yfp were very reduced, 329- and 400-fold, respectively, compared to the WT group (Fig. 3d and Table 1). This quantification is independent of the ability of viruses to reactivate *ex vivo*. Thus, the higher reactivation titers of v-KM.yfp than those of v-kLANA.yfp reflect a higher latency load rather than an *in vitro* reactivation phenotype.

**TABLE 1:** Reciprocal frequency of MuHV-4 infection in total splenocytes^*a*^

Virus	Reciprocal frequency^*b*^ of viral-DNA-positive cells (95% confidence interval)
v-WT.yfp	70 (45–158)
v-kLANA.yfp	1,169 (735–2,854)
v-8A10.yfp	23,040 (14,710–53,150)
v-KM.yfp	250 (160–561)
v-KM8A10.yfp	28,500 (18,240–65,080)

a Data were obtained from pools of 5 spleens for each infection group.

b Determined by limiting dilution analysis.

**FIG 4:**
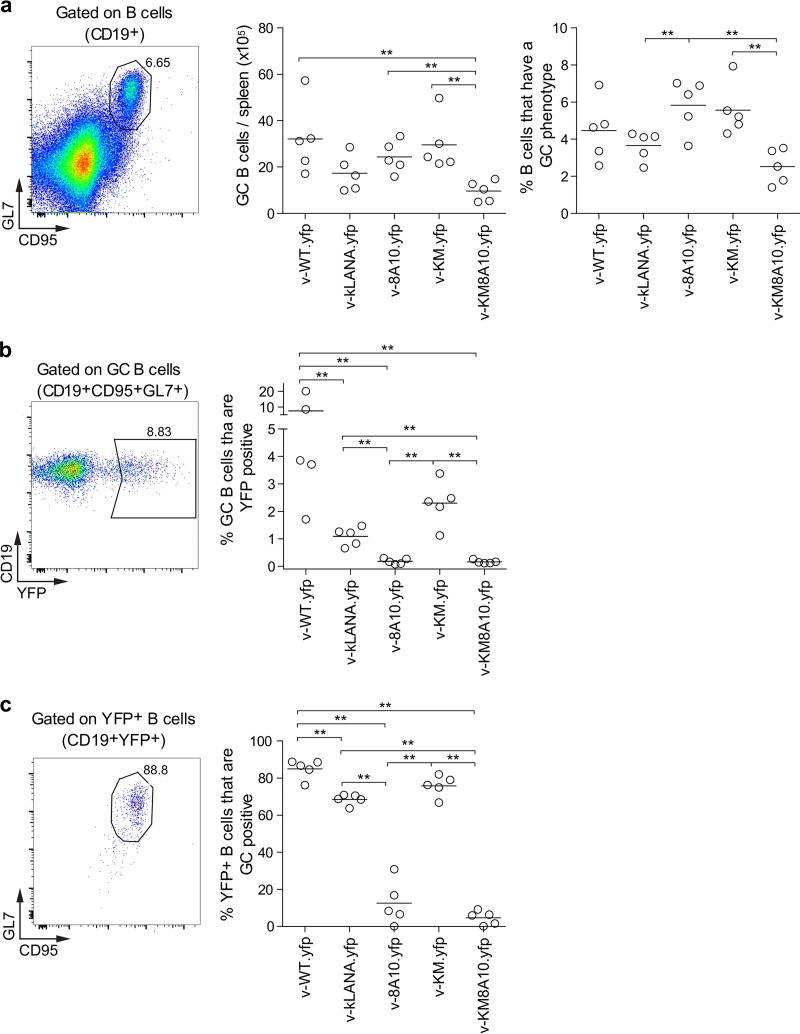
Latent infection in GC B cells. Flow cytometry analysis of splenocytes at day 14 after infection (i.n., 10^4^ PFU). (a) B cells (CD19^+^) with a GC (CD95^+^ GL7^+^) phenotype; (b) GC B cells that were YFP positive; (c) YFP-positive B cells (CD19^+^ YFP^+^) with a GC phenotype. Representative flow plots are shown on the left. Quantification graphs are on the right side of the panels. Each point represents an individual mouse. Horizontal lines indicate means. Statistically significant differences between infection groups are shown (*, *P* < 0.05; **, *P* < 0.01, Mann-Whitney test).

We also assessed infection in GC B cells at day 14 by flow cytometry (Fig. 4a to c). The mean total number and percentage of GC B cells in the different infectious groups varied from 9.7 × 10^5^ to 32.1 × 10^5^ and 2.5% to 5.8%, respectively (Fig. 4a, right panels). YFP expression marked infected cells. The mean percentages of GC B cells expressing YFP were 7.6% for v-WT.yfp, 1.1% for v-kLANA.yfp, and 2.3% for v-KM.yfp (Fig. 4b, right panel). The mean percentages of YFP-positive B cells that had a GC phenotype were 85.0%, 68.5%, and 75.8% for v-WT.yfp, v-kLANA.yfp, and v-KM.yfp, respectively (Fig. 4c, right panel). Significantly reduced percentages of YFP-positive GC B cells were observed for the kLANA v-8A10.yfp and v-KM8A10.yfp viruses compared to other groups (*P* < 0.01) (Fig. 4a and b). Taken together, the data indicate that GC B cells were latently infected with v-WT.yfp, v-kLANA.yfp, and v-KM.yfp.

### Detection of kLANA-C-terminal mLANA fusion protein in the spleen.

**FIG 5:**
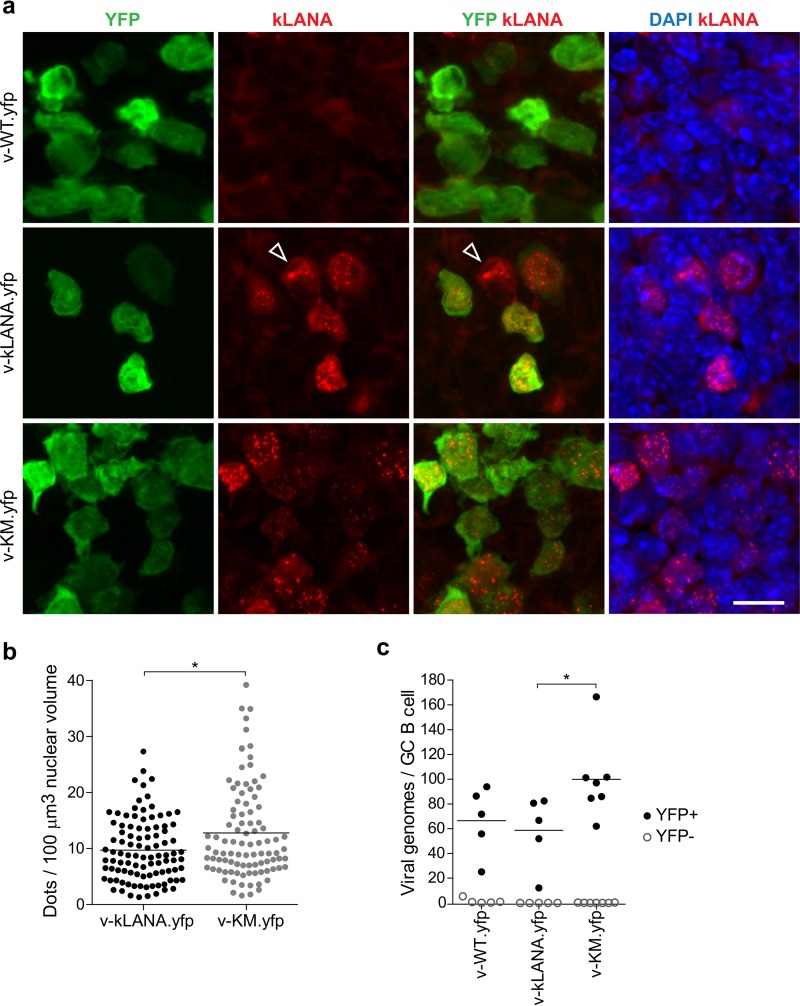
Detection of LANA proteins in infected spleens. (a) Immunofluorescence images (maximal-intensity z-stack projections) depicting YFP- and LANA-expressing cells in spleens of infected C57 BL/6 mice (10^4^ PFU, i.n., day 14). DNA was detected with DAPI. Arrow indicates YFP-negative cell with dots. Bar, 10 μm. (b) Quantification of LANA dots in the nucleus of YFP-positive cells in v-kLANA.yfp-infected (3 mice, *n* = 98) and v-KM.yfp-infected (3 mice, *n* = 95) spleens. Circles represent individual nuclei. Horizontal lines indicate the mean. There was a significant difference between v-kLANA.yfp and v-KM.yfp infection groups (*, *P* < 0.05, Mann-Whitney test). (c) Quantification of viral genome copies per cell in FACS-sorted YFP^+^ (black circles) and YFP^−^ (open circles) GC B cells. Circles represent individual mice. Horizontal lines indicate the means. v-KM.yfp had significantly higher genome copies per cell than did v-kLANA.yfp (*, *P* < 0.05, Mann-Whitney test). No significant differences were found between v-KM.yfp and v-kLANA.yfp and v-WT.yfp (Mann-Whitney test).

To assess expression of the LANA fusion *in vivo*, we performed immunofluorescence assays of spleen sections of infected mice (Fig. 5a). Control v-WT.yfp-infected spleens had many YFP-positive cells and, as expected since they lack kLANA, no staining with anti-kLANA EQEQ MAb LN53 (Fig. 5a, top panels). v-WT.yfp- and v-KM.yfp-infected spleens had higher frequencies of YFP-positive cells than did v-kLANA.yfp-infected spleens (Fig. 5a). YPF-positive cells from v-KM.yfp and v-kLANA.yfp contained nuclear kLANA dots (Fig. 5a, middle and bottom panels). LANA concentrates to dots at sites of viral episomes, and therefore each dot corresponds to a viral genome (16, 21, 41). The intensity of YFP expression varied (Fig. 5a, left panels). Infrequently, YFP-negative cells contained kLANA dots (Fig. 5a, arrow in middle panels). This finding is expected, since loss of YFP expression occurs during latent infection, as shown by PCR detection of viral genomes in YFP-negative cells (reference 42 and data not shown). The number of dots per nuclear volume (100 μm^3^) was slightly higher for v-KM.yfp-infected (mean, 12.8; range, 1.6 to 39.2) than for v-kLANA.yfp-infected (mean, 9.7; range, 1.3 to 21.3) cells (*P* < 0.05) (Fig. 5b). The number of genomes per fluorescence-activated cell sorter (FACS)-sorted YFP + GC B cell was higher in v-KM.yfp (mean, 99.9; range, 62 to 166.5) than in v-kLANA.yfp (mean, 58.8; range, 12.1 to 82.4) (*P* < 0.05) or v-WT.ypf (mean, 66.6; range, 25.1 to 93.9) (statistically not significant) infection groups (Fig. 5c). These data demonstrate that v-KM.yfp and v-kLANA.yfp persist at the WT genome copy number or higher in nuclei of latently infected splenocytes.

## DISCUSSION

In this work, we describe a recombinant MuHV-4 encoding a kLANA (amino acids [aa] 1 to 994)-C-terminal mLANA (aa 118 to 314) fusion protein in place of mLANA. The kLANA fusion protein was detected in the spleens of infected mice and supported latent infection in GC B cells. A different recombinant MuHV-4 encoding kLANA 1 to 982 fused to mLANA 118 to 314, replicated, and expressed the hybrid protein *in vitro* but did not establish detectable latency (data not shown). It is likely that this fusion resulted in folding that disrupted LANA function, perhaps impacting dimerization or oligomerization or hindering the DNA binding ability of the DBD. Thus, the specific fusion site forming the junction between the two proteins is critical for function.

These results indicate that kLANA can accommodate mLANA's strikingly different mode of DNA binding and remain functional. This result was not necessarily expected. mLANA DBD oligomers are linear and rigid versus those of kLANA DBD oligomers, which are bent and can adopt different angles of bend and twist. When complexed with TR DNA, kLANA, but not mLANA, induces conformational change in DNA (19). Given the specific oligomerization mode of the mLANA DBD and the size of the kLANA internal repeat region, ∼600 residues in length, that is absent from mLANA, steric hindrance and a conflict between these two regions in the fusion protein could occur. It is also possible that these different modes of assembly could lead to different functionalities through binding to distinct partners, which could affect transcription or episome replication. Yet, despite the different binding characteristics, the chimeric virus efficiently established latency, indicating a functional chimeric LANA protein.

The latency load of the chimeric v-KM, however, was still lower than that of WT although higher than that of v-kLANA. It is possible that host factors in murine cells may not interact with kLANA regions as efficiently as those from human cells, hampering expansion of cells latently infected with the MuHV-4 expressing the kLANA-C mLANA fusion. It is also possible that kLANA regions could elicit recognition by the mouse immune system, leading to clearance of some virally infected cells.

Both kLANA and the LANA fusion formed LANA dots in the nuclei of infected splenocytes. These dots were similar to mLANA dots that we previously observed in WT-MuHV-4-infected mice (24). Each nuclear dot corresponds to a virus episomal genome, indicating similar genome copy numbers per nucleus for v-kLANA and v-KM. PCR data demonstrated that both v-kLANA and v-KM had similar numbers of genomes per infected GC B cell compared to v-WT. These results are consistent with previous results demonstrating a WT genome copy number in infected splenocyte nuclei for kLANA chimeric virus.

Virus persistence in proliferating, latently infected cells requires episome persistence. Therefore, these data indicate that the kLANA fusion protein is functional for episome maintenance. During episome persistence, LANA tethers viral DNA to mitotic chromosomes to ensure segregation of virus genomes to daughter cell nuclei. Binding to histones is required for LANA attachment to chromosomes. As expected, mutation of the histone binding site of N-terminal kLANA, which abolishes mitotic chromosome association, abolished latency of MuHV-4 expressing full-length kLANA or the fusion protein. This result also highlights the usefulness of the chimera expressing the fusion protein to address *in vivo* kLANA function.

In conclusion, the recombinant MuHV-4 described here demonstrates that kLANA can functionally accommodate the mLANA DBD, despite its innate differences in DNA binding. Further, it importantly provides an alternative model to investigate non-DBD kLANA regions *in vivo*. Because this chimeric virus persists at higher latency levels than v-kLANA, it also provides a greater dynamic range for analysis of phenotypes.

## MATERIALS AND METHODS

### Ethics statement.

Animal studies were performed in accordance with the Portuguese official Veterinary Directorate (Portaria 1005/92), European Guideline 86/609/EEC, and Federation of European Laboratory Animal Science Associations guidelines on laboratory animal welfare. Animal experiments were approved by the Portuguese official veterinary department for welfare licensing (protocol AEC_2010_017_PS_Rdt_General) and the IMM Animal Ethics Committee.

### Cells.

NIH 3T3 CRE (43) cells were grown in Dulbecco's modified Eagle's medium supplemented with 10% fetal bovine serum (FBS), 2 mM glutamine, 100 U/ml penicillin, and 100 μg/ml streptomycin. BHK-21 (C13) cells were grown in Glasgow′s modified Eagle's medium supplemented as described above plus 10% tryptose phosphate broth.

### Antibodies.

Primary antibodies were anti-kLANA rat monoclonal antibody (LN53; ABI Sciences) used at 1:500 to 1:1,000, murine anti-mLANA monoclonal antibody 6A3 (44) used at 1:20 (hybridoma supernatant), and rabbit polyclonal serum against MuHV-4 cyclin (ORF72) and M3 (24) used at 1:500 and 1:3,000, respectively. Rabbit polyclonal antiactin (Sigma) was used at 1:1,000, and a mouse anti-eGFP MAb (where eGFP is enhanced green fluorescent protein; Clontech) was used at 1:1,000. Horseradish peroxidase-conjugated secondary antibodies were from GE Healthcare and Jackson ImmunoResearch. Rabbit polyclonal anti-GFP AF488 antibody (Life Technologies) was used at 1:200, and anti-rat Alexa Fluor 568 (Molecular Probes) was used at 1:250. Antibodies used for flow cytometry were anti-CD19 APC-H7 (clone 1D3; BD Biosciences) used at 1:400, anti-GL-7 eF660 (clone GL7; eBioSciences Inc.) used at 1:200, and CD95 PE (clone Jo2; BD Pharmingen) used at 1:800.

### Viruses.

MuHV-4 (v-WT) was reconstituted from MuHV-4 BAC (45, 46). v-WT.yfp is MuHV-4 expressing the enhanced yellow fluorescence protein (YFP) and was reconstituted from MuHV-4 YFP bacterial artificial chromosome (BAC) (42). v-kLANA and v-8A10, including the yfp versions, have been described (24). To generate the kLANA_1–994_/mLANA_118–314_ fusion protein, a fragment encompassing the C-terminal region of the mLANA coding sequence and downstream genomic regions (GenBank accession U97553, coordinates 102,722 to 104,040) was PCR amplified with primers IMM_TR3 XhoI (5′-AAACTCGAGCAGATGAGATCTGTACTC-3′; BglII site coordinate 102,728 underlined) and mLANA HindIII_A **(**5′-AAAAAGCTTCTGGCACACAACATGGAC-3′; HindIII site coordinate 104,035 underlined). A second PCR-derived fragment containing mLANA coding sequences (coordinates 104,035 to 104,518) attached to the kLANA coding sequence was obtained with primers mLANA HindIII_B **(**5′-AAAAAGCTTGTGTACTTGTGGATGGCTG-3′, HindIII site coordinate 104,035 underlined) and mLANA R994 NruI (5-phosphate-cgaataccgctatgtactcagaacatcaccaccccacagaCCCGGTCCCCCTCCACCAAA-3′; NruI hemisite underlined, kLANA coding sequence in lowercase letters, mLANA coding sequence in uppercase letters). The two PCR products were sequentially cloned using XhoI/HindIII and HindIII/NruI sites into previously described pSP72_PCR1_5 (24) to generate pSP72_ PCR1_5_994. A kLANA fragment encoding the L_8_R_9_S_10_ to A_8_A_9_A_10_ substitutions was excised from pSP72 PCR1/2/3 kLANA_8AAA10_ (24) using BamHI and SacI and cloned into BamHI- and SacI-cut pSP72 PCR1_5_994, to generate pSP72 PCR1_5_994-8A10. The DNA encoding the kLANA-C mLANA fusion proteins plus kLANA 5′ UTR flanked by MuHV-4 genomic sequences was excised with BglII from pSP72 PCR1_5_994 and pSP72 PCR1_5_994-8A10 and subcloned into the BamHI-G MuHV-4 genomic fragment cloned in pST76K-SR shuttle (46), using BglII sites (coordinates 102,728 and 105,087). Each of the mutant BamHI-G shuttle vectors was used for recombination with wild-type MuHV-4 BAC (pHA3) (46) or MuHV-4 YFP BAC (42) in Escherichia coli DH10B. Recombinants were identified by kLANA-specific PCR and BamHI, HindIII, and EcoRI restriction profiles. Viruses were reconstituted by transfection of recombinant BAC DNA into BHK-21 cells using X-tremeGENE HP (Roche). The loxP-flanked BAC cassette was removed by passage through NIH 3T3-CRE cells.

### Infectivity assays.

Viral stocks were prepared by infection of BHK-21 cells (24). Infectious virus titers were determined by plaque assay (suspension assay) in BHK-21 cells. Six- to 8-week-old C57 BL/6J female mice (Charles River) were inoculated intranasally under isoflurane anesthesia with 10^4^ PFU in 20 μl in phosphate-buffered saline (PBS). Lungs or spleens were harvested at the indicated time points. Lungs were homogenized, freeze-thawed, and titrated in BHK-21 cells. To prepare single-cell suspensions, spleens were mechanically disrupted and filtered through a 100-μl cell strainer. Cells were incubated with a hypotonic NH_4_Cl solution for lysis of red blood cells and, after washing, resuspended in 2% fetal bovine serum in PBS for limiting dilution and flow cytometry analysis or resuspended in cell medium for infectivity assays. Reactivating virus was quantified by coculture with BHK-21 cells. Preformed infectious virus was determined by plaque assay in freeze-thawed samples. Plates were incubated for 4 days for plaque assay or 5 days for coculture assay. Cells were fixed with 4% formaldehyde and stained with 0.1% toluidine blue, and viral plaques were counted with a plate microscope.

### Western blotting.

Cells were washed with PBS and disrupted with ice-cold lysis buffer (10 mM Tris-HCl [pH 7.4], 150 mM NaCl, 1 mM NaF, 1 mM orthovanadate, 1% Triton X-100, and complete protease inhibitors from Roche). Lysates were cleared by centrifugation. Approximately 1.25 × 10^5^ uninfected or infected BHK-21 cell equivalents were loaded per lane for protein detection. Proteins were resolved by 10% SDS-PAGE, transferred to nitrocellulose, and probed with the indicated antibodies.

### Quantification of viral transcripts.

Total RNA was extracted from ∼6 × 10^5^ uninfected or infected BHK-21 cells (5 PFU/cell, 8 h) with TRIzol (Invitrogen). Genomic DNA was eliminated by treatment with 2 U of DNase I (Turbo DNase; Ambion). DNase-treated RNA (500 ng) was retrotranscribed using 2.5 μM oligo(dT) primer and 10 U/μl Superscript III reverse transcriptase (Invitrogen) in a 20-μl reaction volume. All samples were treated with 2 U RNase H (Invitrogen), and 2 μl of a 1/10 dilution was used in each quantitative PCR (qPCR; Dynamo Flash SYBR green qPCR kit). Primer pairs, designed with Primer3, were M11_F1 (5′-ACCCAGGAGTTTAGAAGGCA) and M11_ R1 (5′-CAACGAGGTGAAAAGTTTGGAC-3′) for M11, M3_F1 (5′-AACATCAAGCTGACCCCAAC-3′) and M3_R1 (5′-GGGTGTGGACTTCAACTTCC-3′) for M3, ORF63_F1 (5′-GCGCTGACAACGACTCTATT-3′) and ORF63_R1 (5′-ATTTGGGCAGGTGGGGTATA-3′) for ORF63, and GAPDH_hams_F2 (5′-ACCTGCCAAGTATGAGGACA-3′) and GAPDH_hams_R2 (5′-AAGGTGGAAGAGTGGGAGTC-3′) for hamster GAPDH (glyceraldehyde-3-phosphate dehydrogenase). Reactions were performed in triplicate using a Rotor Gene 6000 and analyzed with Rotor Gene 6000 software (Corbett Life Science). ΔΔ*C_T_* values (where *C_T_* is threshold cycle) were calculated. Samples prepared in parallel without addition of reverse transcriptase confirmed that contamination with genomic DNA was negligible (data not shown).

### Flow cytometry.

Flow cytometry using single-cell suspensions prepared from spleens was performed as previously described (24). Fluorochrome-conjugated antibodies against CD19, GL-7, and CD95 were used to identify GC B cells. Data were acquired on an LSR Fortessa (BD BioSciences) with DIVA software and analyzed with FlowJo 9.3.2 (Tree Star). YFP-negative (YFP^−^) and YFP-positive (YFP^+^) GC B cells were sorted using a BD FACSAria flow cytometer (BD BioSciences). The purity of sorted populations was above 98%.

### Frequency of viral-genome-positive cells.

The frequency of viral-genome-positive cells was determined by combining limiting dilution with real-time PCR to detect the MHV68 M9 gene as described previously (47).

### Quantification of viral genomes.

YFP^+^ and YFP^−^ GC B cells were FACS sorted from individual spleens of infected mice (24). Sorted cells were washed with PBS, resuspended in PBS, diluted 1:3 in lysis buffer (10 mM Tris-HCl [pH 8.3], 3 mM MgCl_2_, 50 mM KCl, 0.45% NP-40, 0.45% Tween 20, and 0.5 mg/ml of proteinase K), and incubated overnight at 37°C. After proteinase K inactivation (95°C for 5 min), samples were assessed in duplicate by qPCR for the MHV68 M9 gene or for the cellular ribosomal protein L8 (Rpl8) gene as described previously (24). PCR products were converted to genome copies by comparison to a standard curve of a plasmid harboring M9 or the rpl8. The number of viral gene copies per cell was obtained by dividing the number of M9 copies by one-half the number of Rpl8 copies.

### Immunofluorescence.

Cells adhering to coverslips were fixed with 4% paraformaldehyde–PBS for 20 min at room temperature (RT) and incubated with 20 mM glycine in PBS (15 min, RT). Cells were permeabilized (0.1% Triton X-100–PBS, 5 min), blocked with 10% FBS in PBS (10 min), and incubated with primary antibodies (1 h) followed by incubation with secondary antibodies (30 min), all at RT. Frozen spleen sections were prepared essentially as described previously (48). Spleens were dissected into PBS, fixed with 1% paraformaldehyde, 10 mM sodium periodate, and 75 mM l-lysine in PBS for 24 h at 4°C, and equilibrated in 30% sucrose–PBS for 18 h at 4°C and in 1:1 30% sucrose–optimal cutting temperature (OCT) matrix compound for 3 to 4 h before being frozen in OCT. Longitudinal sections of 10 μm were air dried for 1 h at RT, rehydrated in water and PBS, and incubated with permeabilization/blocking buffer (0.3% Triton X-100, 5% FBS in PBS) for 1 h at RT. Sections were incubated overnight with primary antibodies (4°C) followed by incubation with secondary antibodies (45 min, RT). Stained cells and spleen sections were mounted in Prolong gold antifade reagent with DAPI (4′,6-diamidino-2-phenylindole; Life Technologies). Images were acquired with a Zeiss LSM 710 confocal microscope using a Plan-Apochromat objective of 63× (1.4 oil) plus zoom of 1 (adhering cells) or 3 (spleen sections) using Zen software. Images were processed with Figi (NIH). Nuclear LANA dots were counted as described previously (24) in YFP-positive cells in spleen follicles.

### Statistical analysis.

Statistical evaluation of results was performed with the nonparametric two-tailed Mann-Whitney test or one-way analysis of variance (ANOVA; Kruskal-Wallis test) using Prism software.
